# Add-on benefits of botulinum toxin type A in combination or sequential therapies for scar management: a systematic review and meta-analysis

**DOI:** 10.3389/fpain.2026.1844493

**Published:** 2026-06-11

**Authors:** Yunfei Ma, Wanming Qu

**Affiliations:** Department of Orthopaedics, Yiling Hospital of Yichang, Yichang, Hubei, China

**Keywords:** botulinum toxin type A, combination therapy, meta-analysis, scar management, sequential therapy

## Abstract

**Background:**

Botulinum toxin type A (BoNT-A) has been increasingly incorporated into multimodal scar management strategies as an adjunctive treatment. However, the additive benefits of BoNT-A when used in combination or sequential therapeutic approaches remain unclear due to limited and heterogeneous evidence.

**Methods:**

A systematic review and meta-analysis of randomized controlled trials was conducted in accordance with the PRISMA guidelines. PubMed, Embase, and the Cochrane Library were searched to identify studies evaluating BoNT-A as an add-on intervention in combination or sequential scar management strategies. Eligible studies compared BoNT-A plus standard scar management with control interventions alone. Primary outcomes included validated scar assessment scores, namely the Vancouver Scar Scale (VSS) and the Stony Brook Scar Evaluation Scale (SBSES). Pooled analyses were performed using mean differences (MDs) with 95% confidence intervals (CIs).

**Results:**

Six randomized controlled trials (RCTs) were included. The pooled analysis suggested that BoNT-A add-on therapy significantly improved scar appearance, with a Mean Difference (MD) of −1.80 for the VSS score and 1.46 for the SBSES score. However, this represents low-certainty evidence.

**Conclusion:**

Current low-certainty evidence suggests that BoNT-A may provide a potential adjunctive benefit for improving scar appearance, particularly in mitigating local mechanical tension. However, because these pooled estimates reflect a composite of heterogeneous treatment paradigms, the findings are suggestive rather than confirmatory and strictly exploratory. Clinical application should remain cautious and individualized, pending further high-quality, standardized RCTs.

**Systematic Review Registration:**

https://www.crd.york.ac.uk/PROSPERO/view/CRD420261354679, PROSPERO CRD420261354679.

## Introduction

Scarring is an inevitable consequence of wound healing and represents a common clinical challenge following surgery or trauma (1, 2). Although many scars remain asymptomatic, others may become hypertrophic, widened, or cosmetically unacceptable, leading to functional impairment and significant psychological distress (3–6). The prevention and management of undesirable scars therefore remain an important concern for both patients and clinicians (2, 7). A wide range of therapeutic approaches has been proposed, including silicone-based products, corticosteroid injections, laser therapy, pressure therapy, and surgical revision; however, the clinical outcomes of these interventions are often variable, and no single strategy has been universally accepted as optimal (2, 7–12). As a result, multimodal treatment strategies that combine different therapeutic approaches are increasingly adopted in clinical practice to optimize scar outcomes (2, 7, 10, 12).

Mechanical tension across healing wounds has been recognized as a key factor contributing to pathological scar formation (13–15). Excessive tension can stimulate fibroblast proliferation, promote collagen overproduction, and ultimately result in hypertrophic scarring or scar widening (13–18). Accordingly, therapeutic strategies aimed at reducing wound tension during the early phases of healing have attracted increasing attention (13, 14, 19). Botulinum toxin type A (BoNT-A), a neurotoxin that induces temporary muscle paralysis by inhibiting acetylcholine release at the neuromuscular junction, has emerged as a potential adjunctive treatment for scar management (20–22). Rather than being used as a stand-alone therapy, BoNT-A has most commonly been incorporated into existing scar management protocols, either in combination with standard treatments or as a sequential intervention administered during the perioperative or early postoperative period (20, 21, 23–25). By reducing local muscle activity, BoNT-A may decrease mechanical forces acting on wound edges and thereby facilitate more favorable scar maturation (13, 20, 21, 26).

In recent years, several randomized controlled trials have investigated the use of BoNT-A as an add-on therapy in combination or sequential strategies for scar management in various clinical settings, including postoperative linear scars and facial surgical scars (20, 21, 23–25, 27–31). These studies have suggested that BoNT-A may improve scar appearance, thickness, and pliability when assessed using validated scar evaluation scales. In addition to its mechanical effects, BoNT-A has been reported to exert biological influences on wound healing, such as modulating fibroblast activity and reducing profibrotic signaling pathways (32–34). Despite these promising findings, the available evidence remains fragmented. Most published trials have involved small sample sizes, heterogeneous combination or sequential treatment protocols, and variable follow-up durations, making it difficult to draw definitive conclusions regarding the true add-on benefit of BoNT-A (35, 36).

To date, no comprehensive meta-analysis has systematically synthesized the evidence regarding the add-on effects of BoNT-A when used in combination or sequential therapeutic strategies for scar management (24). As a result, the magnitude of its incremental clinical benefit and the consistency of outcomes across different scar types and anatomical locations remain unclear. A rigorous quantitative evaluation is therefore needed to clarify the role of BoNT-A within multimodal scar management paradigms (35–37).

The objective of the present study was to conduct a systematic review and meta-analysis of randomized controlled trials to evaluate the efficacy of BoNT-A as an adjunctive component of combination or sequential therapies for scar management. Specifically, we aimed to compare scar outcomes between BoNT-A–treated and control groups using validated assessment tools, including the Vancouver Scar Scale (VSS) and the Stony Brook Scar Evaluation Scale (SBSES) (38, 39). By synthesizing the available evidence, this study seeks to clarify the incremental benefits of adding BoNT-A to existing scar management strategies and to inform clinical decision-making and future research.

## Methods

Meta-analysis was carried out in compliance with the Preferred Reporting Items for Systematic Reviews and Meta-analyses (PRISMA)statement. And Our meta-analysis had been registered on the official PROSPERO website with the registration number CRD420261354679.

### Protocol and reporting standards

This systematic review and meta-analysis was conducted in accordance with the Preferred Reporting Items for Systematic Reviews and Meta-Analyses (PRISMA) guidelines. The study aimed to evaluate the add-on benefits of botulinum toxin type A (BoNT-A) when used in combination or sequential therapeutic strategies for scar management.

### Literature search strategy

A comprehensive electronic literature search was performed in the following databases: PubMed, Embase, and the Cochrane Library. A total of 220 records were identified through database searching (PubMed=130, Embase=52, Cochrane=38), and 2 additional records were identified through other sources.

**Figure 1:**
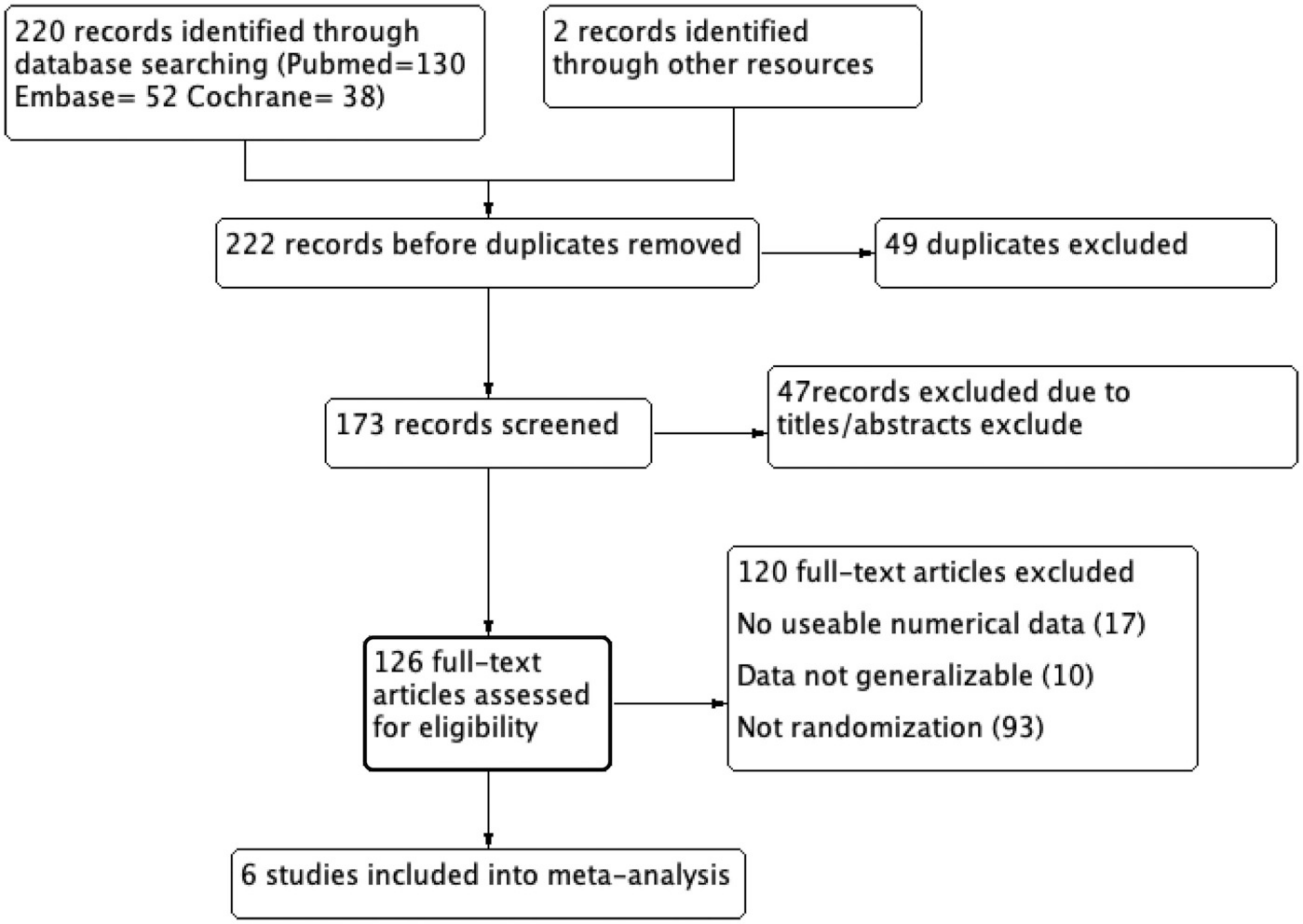
PRISMA flowchart illustrating the study selection, screening, and inclusion process for the systematic review and meta-analysis.

Duplicate records were removed prior to screening. Titles and abstracts were initially screened, followed by full-text review for eligibility. The detailed study selection process is shown in the PRISMA flow diagram (Figure 1). The search utilized Boolean operators and database-specific terms (e.g., MeSH). Full search strings are provided in Supplementary Material S1.

### Inclusion criteria

Eligibility criteria followed the PICOS framework: (P) patients with scars; (I) BoNT-A as an exclusive add-on therapy; (C) identical baseline treatments without BoNT-A; (O) VSS and SBSES scores; (S) pure RCTs. Crucially, “add-on therapy” was strictly defined as applying identical baseline therapies to both arms, isolating BoNT-A as the sole independent variable. Studies were included if they met the following criteria: (1) Study design: randomized controlled trials (RCTs), including parallel-group RCTs or split-scar/split-face randomized self-controlled trials. (2) Population: patients with surgical scars (e.g., post-thyroidectomy, post-sternotomy) or facial postoperative scars (e.g., post-epicanthoplasty medial canthal scars). (3) Intervention: BoNT-A injection used as an add-on treatment, either combined with other scar management approaches or administered in sequential post-treatment strategies (e.g., intraoperative/early postoperative injection). (4) Comparator: placebo or control intervention (e.g., saline injection). (5) Outcomes: studies reporting at least one scar evaluation outcome using validated assessment scales, including: Vancouver Scar Scale (VSS) and Stony Brook Scar Evaluation Scale (SBSES).

### Exclusion criteria

Studies were excluded if: (1) Full texts did not provide usable quantitative data for meta-analysis. (2) The data were not generalizable for effect synthesis. (3) The study design was not randomized. According to the PRISMA flow diagram, full-text exclusions were primarily due to non-randomization, lack of usable numerical data, or non-generalizable outcomes.

### Study selection

After removing duplicates, 173 records were screened by title and abstract. A total of 128 full-text articles were assessed for eligibility. Ultimately, 6 studies were eligible for quantitative meta-analysis (20, 21, 24, 28, 40, 41).

### Data extraction

Data extraction was performed using a standardized data collection form to ensure consistency across included studies. For each eligible trial, we extracted key study characteristics including the first author and year of publication, study design, sample size, scar type, intervention details (including BoNT-A administration strategy and timing), comparator regimen, duration of follow-up, and reported outcome measures. When necessary, numerical outcome data required for meta-analysis were retrieved directly from the text, tables, or figures of the original articles, and all extracted information was cross-checked to minimize errors and ensure accuracy. The overall certainty of evidence for primary outcomes was evaluated using the GRADE framework.

### Risk of bias assessment

**Figure 2:**
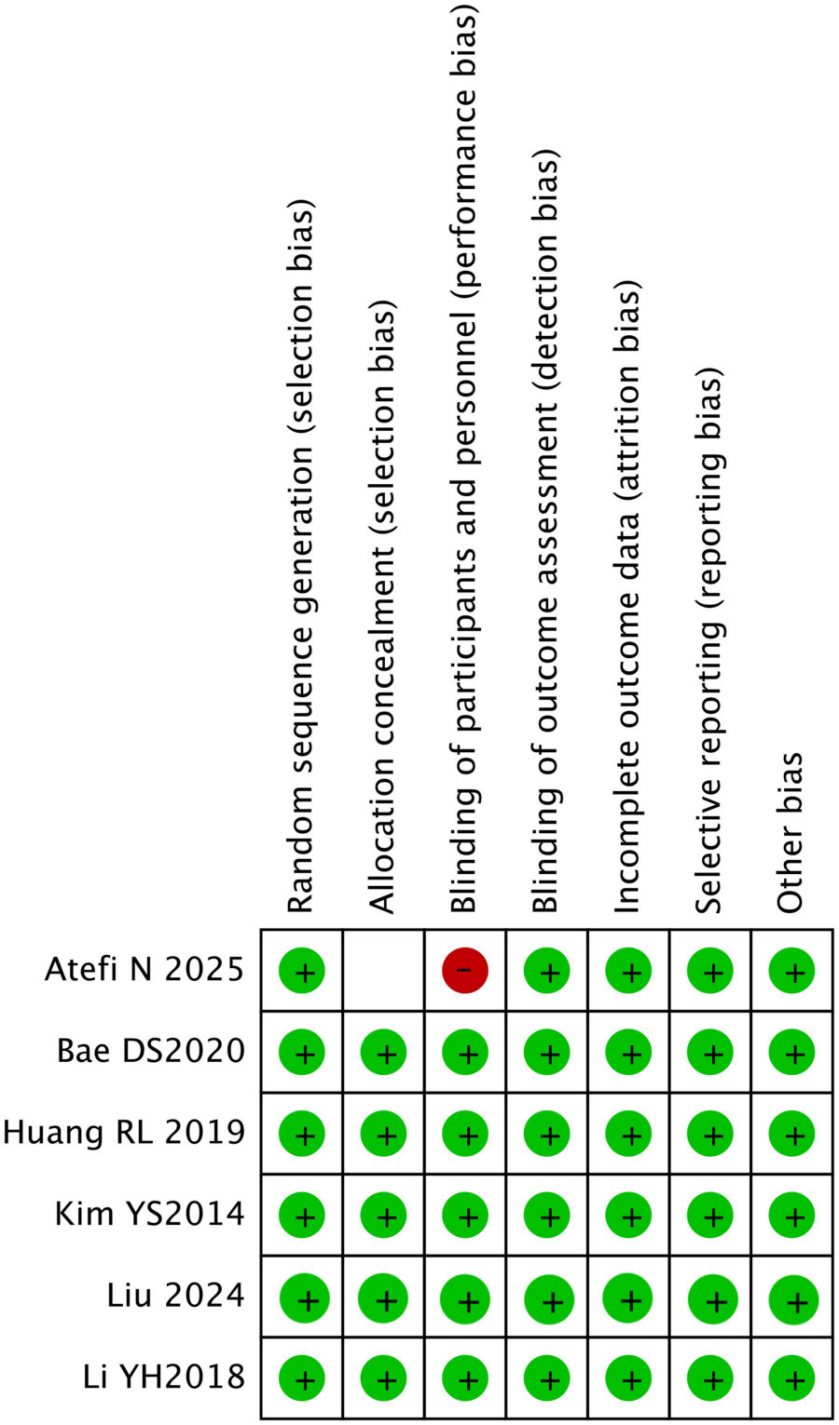
Methodological quality and risk of bias summary graph across the seven bias domains for the six included randomized controlled trials.

The methodological quality of the included randomized controlled trials was assessed using the Cochrane Risk of Bias tool. Risk of bias was evaluated across the following domains: random sequence generation, allocation concealment, blinding of participants and personnel, blinding of outcome assessment, completeness of outcome data, selective reporting, and other potential sources of bias. Each domain was judged as having low risk of bias, high risk of bias, or unclear risk of bias according to the information provided in the included studies. A risk-of-bias summary graph was generated to present the overall methodological quality of the included evidence (Figure 2).

### Outcomes

The primary outcomes of interest in this meta-analysis were validated scar assessment scores, including the Vancouver Scar Scale (VSS) and the Stony Brook Scar Evaluation Scale (SBSES). These outcomes were selected because they are commonly used clinical instruments for evaluating scar severity, incorporating features such as pigmentation, vascularity, pliability, and scar height or appearance. When studies reported outcomes at multiple follow-up time points, the longest available follow-up was preferentially extracted for quantitative synthesis to reflect the sustained effect of add-on BoNT-A therapy.

### Statistical analysis

All continuous outcomes used the same scales and were pooled using the Mean Difference (MD) with corresponding 95% confidence intervals (CIs). To prevent unit-of-analysis errors in split-body designs, the Generic Inverse Variance (GIV) method was applied. Statistical heterogeneity among studies was assessed using the I^2^ statistic, and heterogeneity was considered substantial when I^2^ values were high. A random-effects model was applied in the presence of substantial heterogeneity, whereas a fixed-effect model was considered when heterogeneity was low. Subgroup analyses were conducted to explore potential sources of heterogeneity based on different scar settings and treatment contexts, including postoperative linear scars, and established hypertrophic scars or keloids. The pooled effects were summarized using forest plots.

## Results

### Search results

**Figure 1** illustrated the PRISMA flow chart of the study selection process. A total of 222 records were initially identified through database searching and other resources. After removing 49 duplicates, 173 records were screened by title and abstract, resulting in the exclusion of 47 records. Of the remaining 126 full-text articles assessed for eligibility, 120 were excluded (17 lacked usable numerical data, 10 involved non-generalizable data, and 93 were non-randomized designs). Ultimately, six pure RCTs were included. Baseline equivalence for each trial is detailed in Table 1, demonstrating symmetrical baseline therapies across experimental and control arms.

**Table 1:** Baseline characteristics and clinical study parameters of the six included randomized controlled trials.

Study	Year	Design	Scar type	Baseline treatment(control)	Intervention(add-on)	Control	Sample size	Follow-up	Outcomes
Kim sy	2014	Split-scar, double-blind rct	Post-thyroidectomy linear scar	Standard postoperative care (silicone gel)+saline injection	Standard postoperative care (silicone gel)+bont-a injection	Saline injection	15 patients	Up to 6 months	Sbses
Bae ds	2020	Double-blind, parallel rct	Post-thyroidectomy scar	Conventional management (silicone gel/sheets)+saline injection	Conventional management (silicone gel/sheets)+bont-a injection	Saline injection	40 patients	Up to 24 weeks	Sbses
Huang RL	2019	Split-face, double-blind RCT	Post-epicanthoplasty medial canthal scar	Conventional surgical wound care (Control side)	Conventional wound care+BoNT-A injection (Experimental side)	Saline injection	30 patients	Up to 6 months	VSS
Li YH	2018	Randomized, double-blind RCT	Post-sternotomy scar	Surgical closure alone (Standard suturing)	Surgical closure+BoNT-A injection	Saline injection	17 patients	6 months	VSS
Liu D	2024	Split-face, double-blind randomized self-controlled trial	Post-epicanthoplasty medial canthal scar	Standard postoperative care (Silicone gel)+Saline injection	Standard postoperative care (Silicone gel)+BoNT-A injection	Saline injection	20 patients	Up to 6 months	SBSES
Atefi N	2025	Three-arm, single-blind RCT	Hypertrophic scars and keloids	Standard therapeutic protocol (Specific baseline treatment)	Standard protocol+BoNT-A injection	PDL alone	10 patients	1 month after final session	VSS

BoNT-A, botulinum toxin type A; GIV, generic inverse variance; PDL, pulsed dye laser; RCT, randomized controlled trial; SBSES, Stony Brook Scar Evaluation Scale; SSA, scar scale assessment; VSS, Vancouver Scar Scale.

### Risk-of-bias assessment

Figure 2 illustrated the risk-of-bias assessment of the included studies. Among the included randomized controlled trials, the majority adequately described random sequence generation, while one study did not clearly report the method used for randomization. Allocation concealment was insufficiently described in several trials, with only a minority of studies providing explicit information regarding concealment procedures.

Blinding of participants and personnel was judged to be at high risk of bias in most studies, largely due to the difficulty of blinding injection-based interventions. In contrast, blinding of outcome assessment was adequately addressed in all included trials, with outcome evaluators reported to be unaware of treatment allocation. All studies provided complete outcome data, and none of the included trials showed evidence of selective outcome reporting. No other significant sources of bias were identified across the included studies.

Given the limited number of included randomized controlled trials, formal assessment of publication bias was not performed. Based on the GRADE framework, the overall evidence certainty was rated as low, primarily downgraded due to serious risk of bias (lack of blinding) and high inconsistency (substantial heterogeneity). (See Supplementary Material S2).

## Results of the meta-analysis

### Effect on Vancouver scar scale (VSS)

Three studies reported Vancouver Scar Scale (VSS) scores and were included in the meta-analysis, comprising a total of 114 patients, of whom 57 received add-on botulinum toxin type A (BoNT-A) therapy and 57 received control treatment. All included studies compared BoNT-A used in combination or sequential strategies with placebo or standard scar management alone.

**Figure 3:**
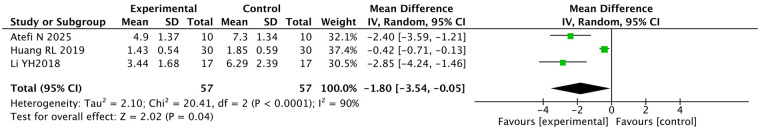
Forest plot displaying the comparison of Vancouver Scar Scale (VSS) scores between the experimental (BoNT-A) and control groups.

Based on a random-effects model, pooled analysis suggested that add-on BoNT-A therapy was associated with a significant improvement in VSS scores compared with the control group [MD = −1.80, 95% CI (−3.54, −0.05), *p* = 0.04; Figure 3]. Overall, patients treated with BoNT-A showed scar improvements compared with those in the control group.

Substantial heterogeneity was observed among the included studies (I^2^ = 90%). To explore potential sources of heterogeneity, subgroup analyses were performed according to scar type and treatment context. These subgroup analyses are exploratory and hypothesis-generating, suggesting a potential adjunctive role of BoNT-A across different clinical scenarios.

### Effect on stony brook scar evaluation scale (SBSES)

Three studies reported outcomes assessed using the Stony Brook Scar Evaluation Scale (SBSES) and were included in the quantitative synthesis. To rigorously prevent unit-of-analysis errors from split-body designs, data were pooled using the Generic Inverse Variance (GIV) method.

Pooled analysis using a random-effects model showed that add-on BoNT-A therapy was associated with a significantly higher SBSES score compared with control treatment [MD = 1.46, 95% CI (0.55, 2.38), *p* = 0.002], indicating a robust visual enhancement in scar appearance for patients receiving BoNT-A.

**Figure 4:**
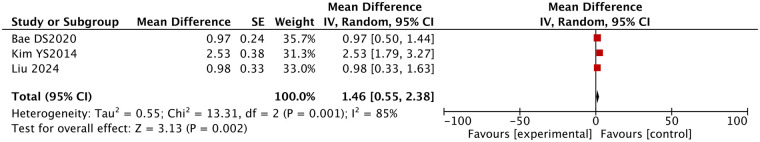
Forest plot displaying the comparison of Stony Brook Scar Evaluation Scale (SBSES) scores between the experimental (BoNT-A) and control groups.

Substantial heterogeneity was observed among the included studies (I^2^ = 85%). Despite the presence of heterogeneity, all included trials generally favored add-on BoNT-A therapy over control treatment in terms of SBSES improvement (Figure 4).

### Subgroup analysis of VSS

Subgroup analyses were performed according to scar type, including postoperative linear scars and established hypertrophic scars or keloids.

In the subgroup of postoperative linear scars, two studies involving 94 patients were included. The pooled analysis showed that add-on BoNT-A therapy was not associated with a statistically significant improvement in VSS scores compared with the control group [MD = −1.54, 95% CI (−3.91, 0.84), *p* = 0.20]. Substantial heterogeneity was observed within this subgroup (I^2^ = 91%).

In the subgroup of established hypertrophic scars or keloids, one study comprising 20 patients was analyzed. The pooled results similarly suggested a statistically significant improvement in VSS scores with add-on BoNT-A therapy compared with control treatment [MD = −2.40, 95% CI (−3.59, −1.21), *p* < 0.0001]. Importantly, given the limited number of studies included in each comparison, all subgroup findings for both VSS and SBSES should be considered strictly exploratory and hypothesis-generating.

**Figure 5:**
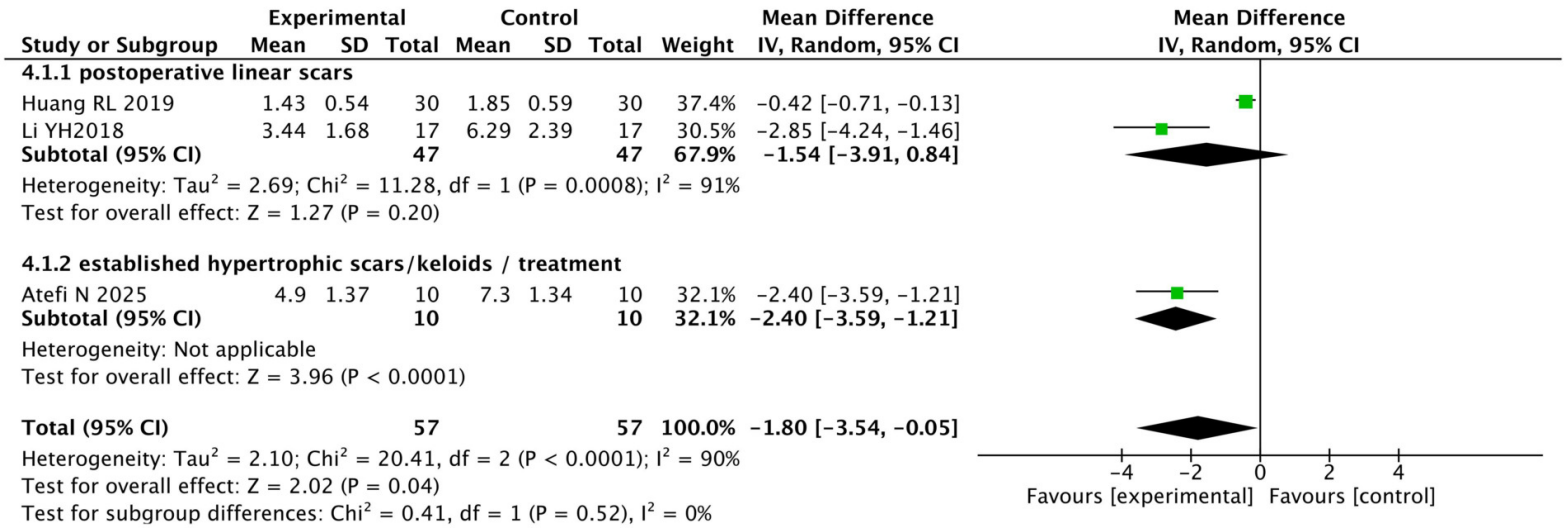
Subgroup analysis of Vancouver Scar Scale (VSS) scores, stratified by scar type (postoperative linear scars vs. established hypertrophic scars/keloids).

While these exploratory findings suggest a potential therapeutic benefit across different scar types, the limited number of studies per subgroup means these results should be interpreted as hypothesis-generating rather than confirmatory (Figure 5).

### Subgroup analysis of SBSES

Subgroup analyses for SBSES outcomes were conducted based on anatomical location, including neck/thyroidectomy scars and epicanthus scars.

For neck/thyroidectomy scars, two studies were pooled. The analysis suggested that add-on BoNT-A therapy resulted in a significantly higher SBSES score compared with control treatment [MD = 1.72, 95% CI (0.19, 3.25), *p* = 0.03]. Substantial heterogeneity was observed within this subgroup (I^2^ = 92%).

For epicanthus scars, one study was included. The results showed a significant improvement in SBSES scores favoring the BoNT-A group [MD = 0.98, 95% CI (0.33, 1.63), *p* = 0.003].

**Figure 6:**
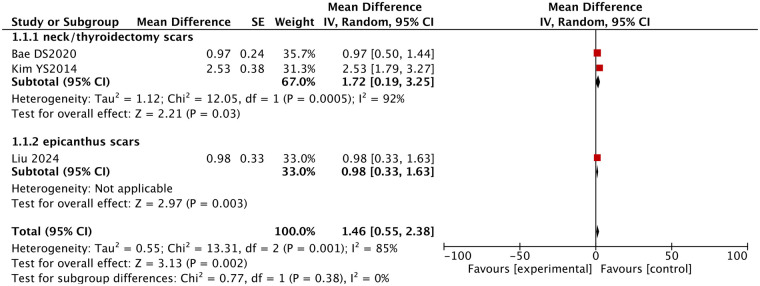
Subgroup analysis of Stony Brook Scar Evaluation Scale (SBSES) scores, stratified by anatomical location (neck/thyroidectomy scars vs. epicanthus scars).

Differences in local mechanical tension between anatomical locations accounted for a substantial portion of the heterogeneity (test for subgroup differences: I^2^ = 51.3%), indicating that scar location inherently modulates the magnitude of BoNT-A’s efficacy (Figure 6).

### Sensitivity analysis

Sensitivity analysis for the VSS outcome (Supplementary Figure S1) was performed by sequentially removing individual studies from the meta-analysis. The overall pooled effect estimates remained stable and statistically significant, suggesting that the statistical computation is not driven by any single trial.

## Discussion

In this systematic review and meta-analysis, we comprehensively evaluated the add-on effects of botulinum toxin type A (BoNT-A) when used in combination or sequential strategies for scar management. The pooled results suggested that adjunctive BoNT-A therapy was associated with significantly improved scar outcomes compared with control treatments. Specifically, BoNT-A significantly reduced Vancouver Scar Scale (VSS) scores [MD = −1.80, 95% CI (−3.54, −0.05), *p* = 0.04] and significantly increased Stony Brook Scar Evaluation Scale (SBSES) scores [MD = 1.46, 95% CI (0.55, 2.38), *p* = 0.002].

It is critical to acknowledge that the pooled estimates in this meta-analysis reflect a composite of markedly heterogeneous treatment paradigms, rather than a uniform biological or clinical effect. Given the high statistical heterogeneity (I^2^ up to 90%), our findings cannot be considered robust across all settings. Consequently, the observed add-on effects of BoNT-A should be interpreted as highly context-dependent and exploratory, and generalizable clinical conclusions are not supported at this stage.

Several biological mechanisms may explain the beneficial effects of BoNT-A. Mechanical tension across healing wounds is a well-recognized contributor to hypertrophic scarring and scar widening. BoNT-A reduces muscle activity adjacent to wounds, thereby decreasing tension at the wound edges during the critical phases of healing. In addition, experimental studies have suggested that BoNT-A may inhibit fibroblast proliferation, suppress excessive collagen deposition, and modulate profibrotic signaling pathways.

Substantial heterogeneity stems from anatomical and scar-type variations. BoNT-A shows pronounced efficacy in high-tension areas (e.g., neck, SBSES MD = 1.72) by offloading mechanical stress (Figure 6). Biologically, early postoperative injection acts as a prophylactic mechanomodulator, whereas delayed injection for established scars primarily targets hyperactive fibroblasts.

When interpreting these results, absolute clinical relevance must be considered. An absolute SBSES improvement of 1.46 points (on a strict 5-point scale) represents robust visual enhancement. Conversely, a 1.80-point reduction in VSS (on a 13-point scale) indicates a more modest clinical benefit, highlighting the future need for Patient-Reported Outcome Measures (PROMs).

From a clinical perspective, incorporating BoNT-A into multimodal or sequential scar management strategies may help optimize cosmetic outcomes. However, given the heterogeneity in treatment protocols, clinical application should be individualized, considering scar characteristics, treatment timing, and patient preferences. Furthermore, a critical clinical limitation of our overall pooled analysis is the combination of two fundamentally distinct scenarios: the prevention of postoperative linear scars and the treatment of established hypertrophic scars and keloids. These scenarios represent completely different biological paradigms, with prophylactic mechanical offloading during early wound healing on one hand and therapeutic metabolic modulation of existing fibrotic tissue on the other. Pooling their estimates artificially merges distinct mechanisms. Consequently, the overall pooled effect must be interpreted with extreme caution. The true clinical value of our findings lies strictly within the exploratory subgroup analyses that separate these preventative and therapeutic applications. However, given the limited number of studies included in each comparison, these subgroup findings should be considered strictly exploratory and hypothesis-generating.

This study has critical limitations. Most notably, the lack of participant and assessor blinding in several trials introduces high performance and detection biases for subjective scales (VSS/SBSES). Evaluator expectancy bias may overestimate BoNT-A's true additive efficacy, justifying our “low” GRADE rating. Furthermore, limited RCT availability (*n* = 6) precluded subgrouping by specific dosing, timings, or distinct baseline therapies (e.g., lasers), which remain vital areas for future large-scale trials.

Future well-designed randomized controlled trials with larger sample sizes are warranted to further clarify the role of BoNT-A. In particular, strictly double-blinded studies should aim to determine the optimal timing, dosage, and injection techniques for BoNT-A, as well as to adopt standardized patient-reported outcome measures and longer follow-up periods. Such efforts would help strengthen the evidence base and refine clinical recommendations.

## Conclusion

In conclusion, current low-certainty evidence suggests that BoNT-A may provide a potential adjunctive benefit for improving scar appearance, particularly by effectively acting as a mechanomodulator to offload stress in high-tension regions. However, it must be explicitly acknowledged that these pooled estimates reflect a composite of markedly heterogeneous treatment paradigms rather than a uniform clinical effect. Given the high statistical heterogeneity, these findings should be considered context-dependent, suggestive rather than confirmatory, and remain strictly exploratory at this stage. Consequently, clinical application should be highly cautious and individualized. Further high-quality, rigidly standardized RCTs are required before firm, generalizable clinical recommendations can be made.

## Data Availability

The original contributions presented in the study are included in the article/Supplementary Material, further inquiries can be directed to the corresponding author/s.
