# Supplementary figures and images for: Add-on benefits of botulinum toxin type A in combination or sequential therapies for scar management: a systematic review and meta-analysis
Source: Front Pain Res (Lausanne). 2026 Jun 11;7:1844493. doi: 10.3389/fpain.2026.1844493 (PMC13293821; doi:10.3389/fpain.2026.1844493)

**the complete search strategy is provided below:**


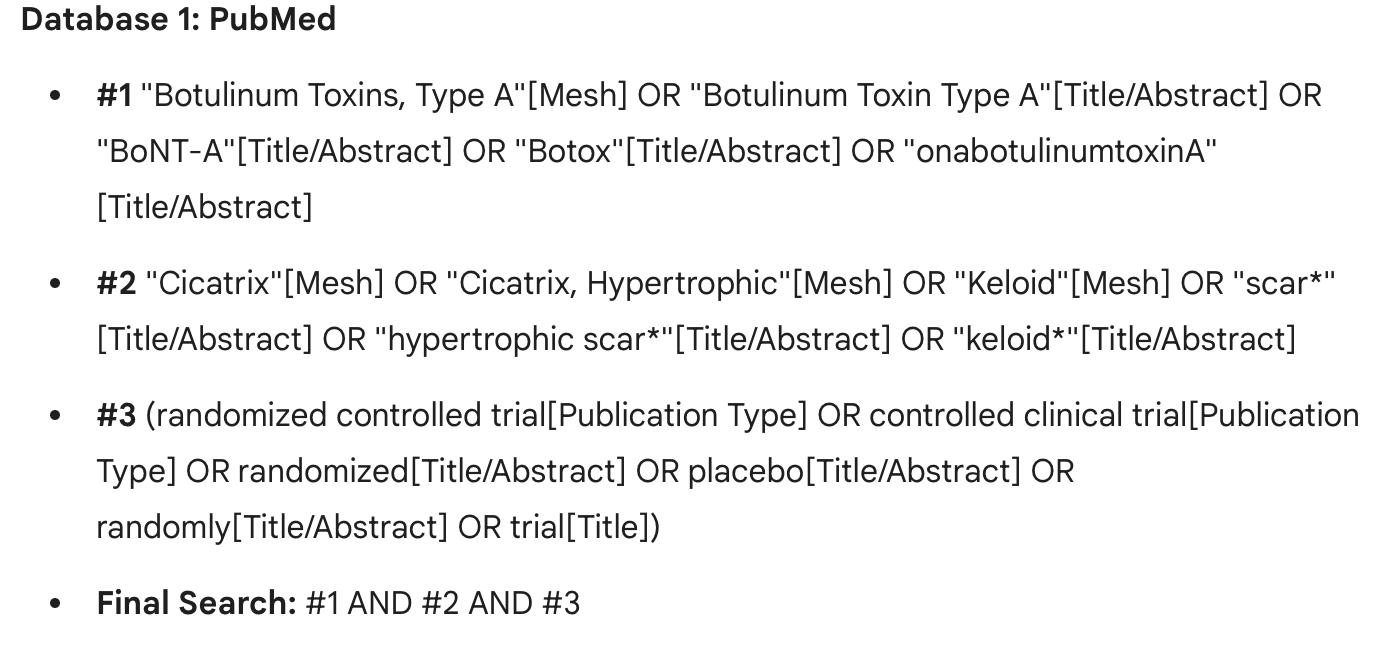

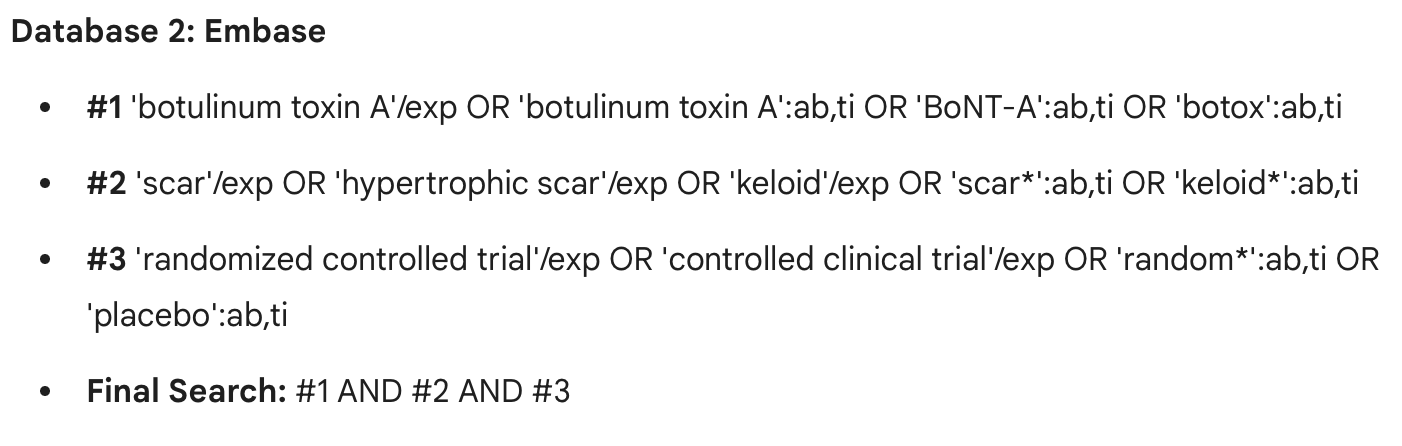

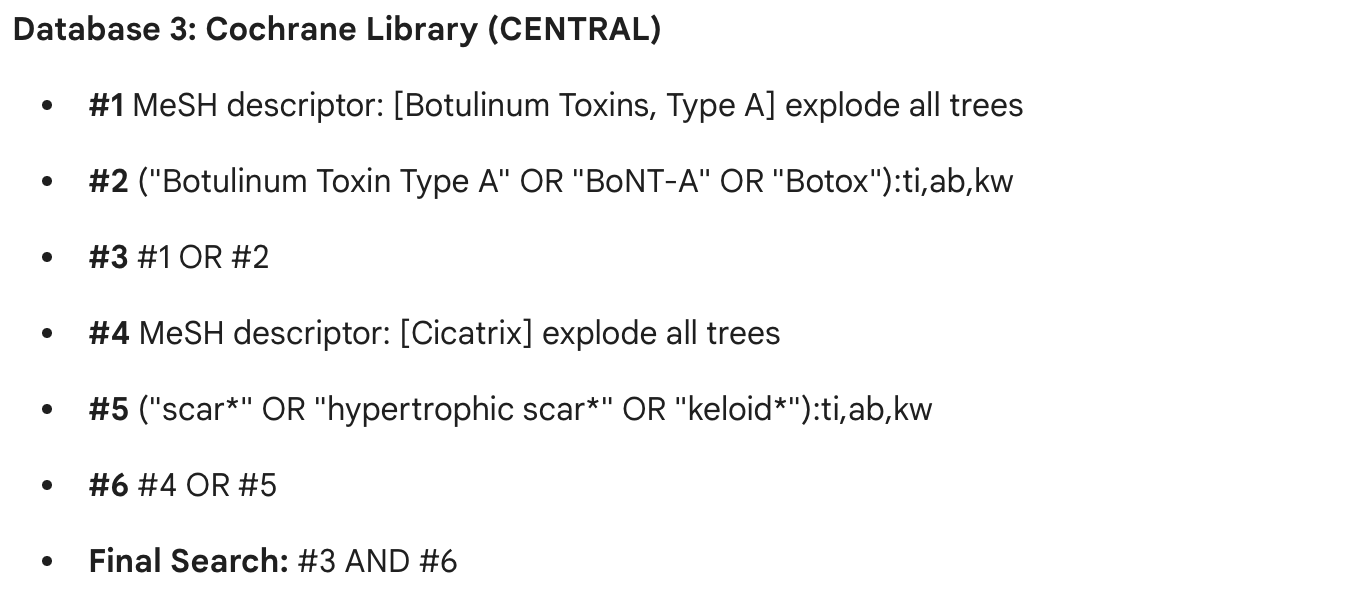

Supplement: Supplementary file 1 [file Table1.docx]

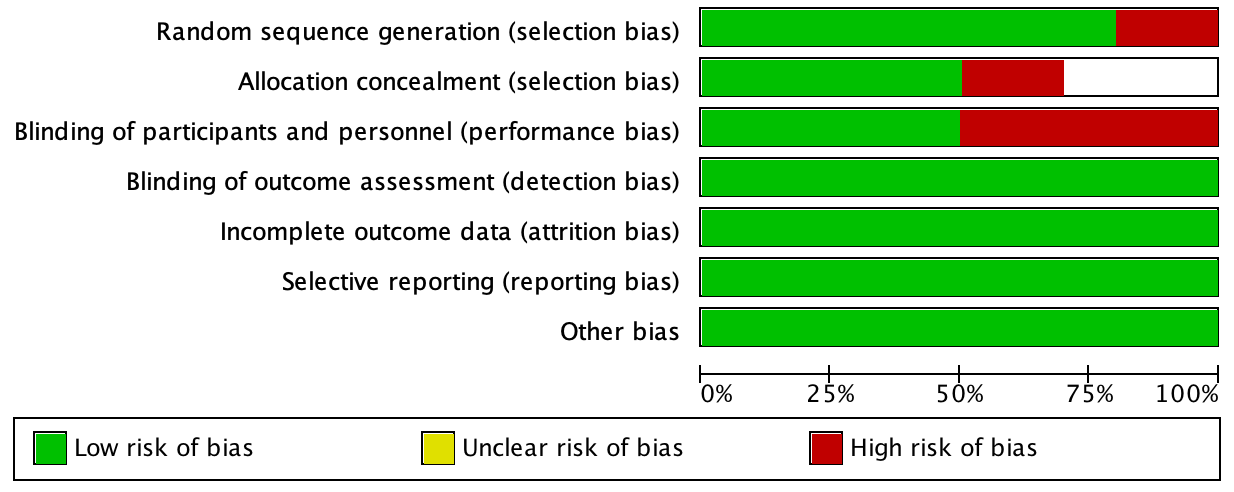

Supplement: Supplementary file 4 [file Image1.png]
